# High-Throughput Solid Phase Extraction for Targeted and Nontargeted Exposomics

**DOI:** 10.1021/acs.analchem.4c06177

**Published:** 2025-03-13

**Authors:** Yunyun Gu, Max Lennart Feuerstein, Benedikt Warth

**Affiliations:** † Faculty of Chemistry, Department of Food Chemistry and Toxicology, University of Vienna, 1090 Vienna, Austria; ‡ University of Vienna, Vienna Doctoral School of Chemistry, Währinger Straße 42, 1090 Vienna, Austria; § Exposome Austria, Research Infrastructure and National EIRENE Node, 1090 Vienna, Austria

## Abstract

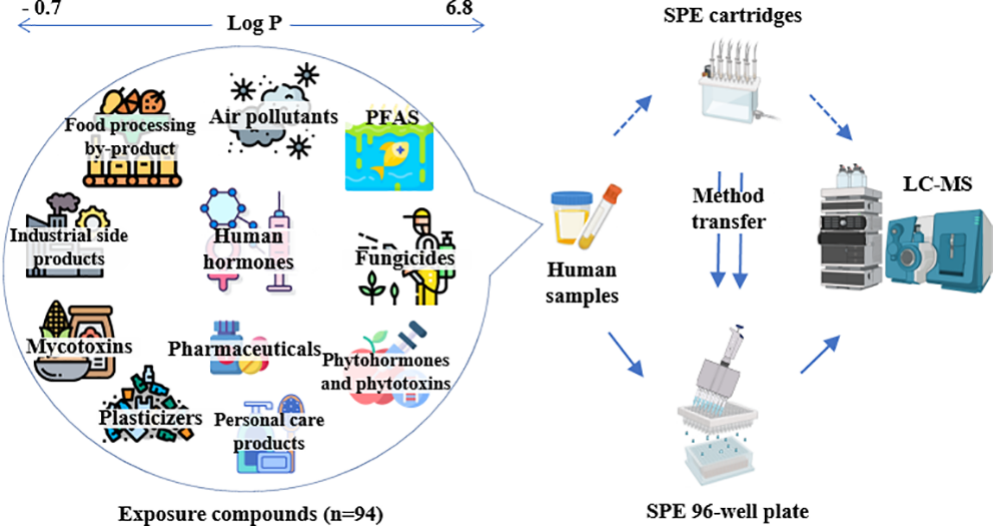

Characterizing the chemical exposome relies on advanced instrumentation including tandem mass spectrometry coupled to liquid chromatography (LC-MS/MS), and nontargeted analysis (NTA) using high-resolution MS. However, proper sample pretreatment, balancing broad analyte coverage, method robustness, and throughput remain a major bottleneck in exposomics. Here, we developed a robust and scalable solid phase extraction (SPE) protocol for human urine and plasma and optimized it for a panel of 94 highly diverse environmental and food-related contaminants (LogP −0.7 to 6.8). Extraction recoveries (RE) and signal suppression and enhancement (SSE) were determined using targeted LC-MS/MS. Acceptable REs (60–140%) were achieved for >70% of analytes, and acceptable SSE values (60–140%) for 86% and 90% in urine and plasma, respectively. Subsequently, the method was transferred to 96-well plate format, significantly improving throughput to meet the capacity requirements needed for exposome-wide association studies (ExWAS). The established workflow is approximately 10× faster than routinely used metabolomics-based protein precipitation approaches when comparing the estimated total analysis time for 1000 samples. The method’s applicability for NTA and suspect screening was tested and compared to a generic protein precipitation protocol using NIST standard reference materials for urine (SRM 3672) and plasma (SRM 1950). Favorable performance was shown for the protein precipitation workflow while the SPE protocol demonstrated promising results. The developed workflow is thus not only superior for future high-throughput targeted exposomics but also offers an option for NTA applications. The presented well-balanced approach is scalable and also applicable to research in the fields of pharmacology, food safety, and systems toxicology.

Humans are consistently exposed to a myriad of chemicals during their daily lives through various sources, including dietary intake and environmental pollutants.^1−3^ Exposomics represents the comprehensive analysis of all environmental and food-related exposures and associated influences on various health outcomes.^4^ To allow a full assessment of the totality of chemical exposures in human samples, sensitive, specific, and robust analytical techniques, such as liquid chromatography-tandem mass spectrometry (LC-MS/MS) are required. Multiclass or next-generation human biomonitoring (HBM) methods have recently been developed to monitor a wide range of environmental chemicals and are needed to address the full complexity of chemical exposure.^5,6^ In contrast, nontargeted analysis (NTA) is an emerging technique for exposomics using high-resolution mass spectrometry (HRMS) and allows the exploration of unknown chemicals in human samples.^7,8^ Furthermore, suspect screening methods use large chemical databases and spectral libraries to prioritize detected features and annotate compound identities.^9,10^ Both targeted and nontargeted exposomic methods face significant challenges related to complex biological sample matrices,^11^ and ensuring repeatability and consistency across large sample sets is mandatory, especially when dealing with low-abundance analytes (i.e., pg/mL levels or lower).^12−14^ Addressing these challenges in exposomics requires sophisticated optimization of the entire analytical workflow, including sample preparation protocols. Efficient sample pretreatment before instrumental analysis can reduce interferences, separate, and concentrate analytes in diverse matrices^15,16^ and various sample preparation techniques have been optimized to analyze the exposome. Liquid extraction (LE) and protein precipitation (PPT) are widely used extraction methods for exposomics due to their comparably wide chemical coverage.^6,17^ However, these techniques are prone to preserve matrix interferences which can hamper overall method performance due to limited sample cleanup.

Recently, solid phase extraction (SPE) received more attention as an option for sample preparation in exposomics and is discussed as a promising approach for future large-scale applications, owing to its capability to reduce matrix effects, enhance sensitivity, and improve consistency in high-throughput studies.^18,19^ SPE has been frequently used for the purification and extraction of various analytes from diverse sample matrices for HBM including urine,^20^ blood,^21^ and various other biological matrices.^22,23^ However, a systematic development of an exposome-scale SPE method compatible with both, targeted and nontargeted data acquisition strategies has rarely been performed. This challenge originates from the complexity of designing a balanced method with suitable method robustness and broad chemical space coverage. Most existing SPE methods focused on a limited selection of targeted analytes to obtain reliable protocols without assessing the potential coverage for NTA^24^ due to the lack of certified reference standards, which restricts their utility in NTA.

In this study, a targeted high-throughput sample pretreatment method using SPE cartridges was optimized to ensure the applicability of the workflow for urine and plasma samples. The predicted octanol–water partition coefficient (logP) from PubChem (pubchem.ncbi.nlm.nih.gov) was utilized to evaluate the chemical coverage in terms of analyte lipophilicity of compounds in this study. Target analytes with a wide range of physicochemical properties, including polarity, presents a central challenge for SPE methods in exposomics, due to varying analyte-sorbent interactions. This issue is a key factor that needs to be addressed during method development aiming for a comprehensive chemical coverage. Here, 94 highly diverse analytes from multiple toxicant classes including perfluorinated substances (PFAS), plasticizers, personal care products, food processing byproducts, pesticides, industrial chemicals, air pollutants, disinfecting agents, mycotoxins, phytoestrogens and phytotoxins were used to optimize an SPE protocol for high-throughput applications. Optimization steps included selecting SPE sorbents and sample buffers, optimizing washing and elution steps, and improving efficiency. Subsequently, the protocol was transferred to 96-well plates, and analytical performance and throughput capacity were rigorously tested. Finally, the potential for NTA was explored using NIST standard reference materials (SRMs) for urine and plasma. The results indicate the feasibility of the approach for scaling and large-scale exposome-wide association studies.

## Experimental Section

### Biological Samples and Chemicals

Human pooled plasma was purchased from Innovative Research (Novi, MI). Pooled urine was obtained from a female volunteer who abstained from consuming food and beverages stored in plastic containers, phytoestrogens rich food, and cosmetics containing parabens for 2 days before sample collection.^14^ Standard reference materials, including urine (SRM 3672) and plasma (SRM 1950) were obtained from the National Institute of Standards and Technology (NIST, Gaithersburg) for NTA. Detailed information on standards, including selection criteria and spiking levels can be found in Tables S1–S4. The selection of target analytes and analyte classes was intended to be as broad as technically feasible with differing structures, real-life exposure levels, and toxicological modes of action. It was based on our previous work on next-generation biomonitoring.^6^

### Method Development and Optimization

The sample pretreatment approach was developed and optimized in several steps. Initially, two universal SPE sorbents, a commercial hydrophilic–lipophilic balanced sorbent (HLB) from Waters and an in-house prepared mixed-mode SPE sorbent, were compared. The mixed-mode sorbent was prepared from two SPE sorbents, primary second amine (PSA) and C18 (PSA+C18), by mixing equal weights before packing. Details of the preparation are described in the Supporting Information (SI). Packed SPE cartridges (HLB or PSA+C18) were then used to optimize the full processes including sample loading, washing, and elution. A mixture of 94 reference standards was spiked into urine, plasma, and water (H_2_O) samples both before (“prespiked”) and after (“postspiked”) the SPE process. Analyte loss (%), extraction recovery (RE, %), and signal suppression and enhancement (SSE, %) were calculated based on the peak areas from these “prespiked” and “postspiked” samples.

Analyte loss was defined as the fraction of an analyte that was not retained by the SPE sorbent. It was calculated as the ratio of peak areas of the effluents collected during sample loading and washing from “prespiked” samples compared to peak areas of “postspiked” effluents. Analyte loss of 0% was achieved for complete analyte retention, whereas completely unretained analytes are characterized by a loss of 100%.

The term extraction recovery (RE) was used to describe the recovery of the full SPE process including loading, washing and elution. Thus, RE was calculated as the ratio of peak areas of a “prespiked” and “postspiked” sample. Full analyte recovery results in RE of 100%, whereas 0% indicates no analyte recovery of the SPE procedure. RE greater than 100% indicate contamination or carryover introduced during sample preparation or sample injection.

Matrix effects in terms of signal suppression and enhancement (SSE) depend on the influence of the sample matrix on the analyte’s signal intensity. This was calculated as the ratio of peak areas for “postspiked” samples to a standard in pure solvent.^25^ SSE values close to 100% indicate negligible effects of the sample matrix on the detector response, which is required for accurate analyte quantification.

To evaluate the method performance and provide a rationale for the individual optimization steps, the metrics described above were classified into three categories: “good”, “acceptable”, and “poor”, as shown in Table S5. Noteworthy, they do not represent method validation criteria but are used for method optimization.

Subsequently, the SPE method was applied to pooled human urine and plasma samples to test applicability to analyze human samples. Sample pretreatment was further streamlined and transferred from SPE cartridges to 96-well plates to allow parallel sample extraction and maximize sample throughput. Furthermore, the applicability of the developed workflow for NTA and suspect screening was tested to further expand the coverage of the targeted biomonitoring assay.

#### SPE Sorbent Selection

SPE cartridges with two different sorbent types, HLB and PSA+C18, were considered and the stability and robustness of the material were tested. For this purpose, loading, washing, and elution procedures were individually evaluated. In this first evaluation, analytical standards in LC-MS grade H_2_O were used as samples to minimize influences of the sample matrix. Analyte loss (%) for both SPE sorbents was tested using H_2_O or 2% methanol (MeOH/H_2_O, 2/98, v/v) for sample loading and washing. Subsequently, RE was assessed using various elution solvents including 3% formic acid in methanol (FA/MeOH, 3/97, v/v), 3% ammonia in MeOH (NH_3_/MeOH, 3/97, v/v), and pure MeOH. Details including conditioning of SPE sorbents, analyte spiking procedures and spiking levels, as well as calculations of the analyte loss and RE, are reported in the SI.

#### Sample Buffer Selection before Loading

Urine and plasma are commonly used sample matrices for HBM and, especially in the case of urine, varying concentrations of matrix components can influence analyte extraction. To ensure method reliability, commonly utilized buffers, PBS (pH 7.4, 200 mM) and NH_4_AC (pH 6.0, 2.5 M), and pure H_2_O were tested for sample dilution before the SPE cleanup. Based on the results of our previously published work, a final dilution ratio of 2 was used.^26^

#### Optimization of Washing, Elution, and Reconstitution Steps

Water was selected as the washing solvent and the required volume was optimized by comparing the SSE of either washing one or two times with 1 mL. MeOH was selected as the primary component of the elution solvent due to its widespread application in SPE processes.^27^ Considering the strong anion-exchange interactions of PSA with commonly encountered acidic compounds in exposomics,^9,28^ a basic MeOH solution (3% NH_3_) was used as the elution solvent to compare the RE of the mixed-mode sorbent (PSA+C18). For HLB, both acidic (3% FA) and basic MeOH (3% NH_3_) were evaluated due to the anion and cation exchange properties of HLB. Furthermore, MeOH and two commonly used solvents, acetonitrile (ACN) and isopropanol (ISO), and a mixture of MeOH/ACN/ISO (1/1/1, v/v/v), were evaluated as elution solvents. The solvent mixture was selected based on results obtained from urine samples. Elution volumes of 200 μL, 400 μL, and 800 μL were subsequently compared using pooled urine as a matrix.

A typical approach to improve detection limits is to evaporate raw extracts and reconstitute them in a defined volume. Despite its clear benefits for enhancing the detection of low-abundant analytes, this step is rather time-consuming, especially when dealing with a large number of samples. To further enhance sample throughput and assess the effect of evaporation and reconstitution, two workflows were tested and compared. One set of SPE extracts was dried using a CentriVap Vacuum concentrator (Labconco) and reconstituted with 50% MeOH (MEOH/H_2_O, 1/1, v/v). In contrast, a second set of extracts was diluted with H_2_O, resulting in a final mixture of 50% MeOH for comparison. The final concentrations in the extracts of both sets were the same.

#### Method Transfer to 96-Well Plates

After the optimization, pooled urine and plasma were used to test extraction and cleanup using the developed workflow for SPE cartridges. Then, the method was transferred to a 96-well plate SPE packed with HLB (Waters Corporation) and the following parameters: SPE plates were conditioned with 1 mL of MeOH, followed by 1 mL of H_2_O. Then, 400 μL of urine or plasma sample were mixed with 400 μL of PBS. The mixture was loaded on the SPE plate and washed twice with 1 mL of H_2_O each. Gravity flow was selected for sample loading, washing and analyte elution. Analytes were eluted using two times 200 μL of MeOH, resulting in a total volume of 400 μL. To ensure reproducible elution, residuals of the eluents were collected by applying negative pressure using a vacuum manifold. RE and SSE were calculated and compared to results determined for SPE cartridges to prove applicability for high-throughput applications.

#### Assessing the Potential of SPE for NTA and Suspect Screening

SPE is a promising approach to enhance method performance for targeted applications, but systematic testing for evaluating chemical coverage is a necessity before any potential application in nontargeted analysis. The coverage of other typically detected analytes in human samples and the effect on signal intensities were evaluated by comparing results of the novel 96-well plate-based SPE approach to results from a PPT method^29^ as the reference method for nontargeted applications. SRM 1950 (plasma) and SRM 3672 (urine) were extracted with both workflows and were analyzed using LC-HRMS for NTA. Unknown compounds were annotated using publicly available spectral libraries and a set of 234 authentic analytical standards (Table S2) was spiked into samples for compound identification and to test the method for targeted screening. Results were reported based on established reporting schemes, with identified compounds reported with the highest confidence level (“level 1”: identified based on authentic chemical standards). Therefore, standards were spiked into extracts from the SPE process. Spiking levels of these standards are reported in Table S4. Following annotation, the effect of the sample preparation method on relative feature intensities was compared. Results were further investigated as a function of retention times and classifications of the annotated features. Details are outlined in the SI.

### Data Acquisition

#### Targeted LC-MS/MS

Front-end LC separation was performed using our previously published method.^6^ Identical separation gradients were used for LC-MS/MS and LC-HRMS(/MS) using an Agilent 1290 Infinity II and a Thermo Scientific Vanquish Horizon, respectively. An Acquity HSS T3 column (1.8 μm, 2.1 × 100 mm, Waters) was used with gradient elution using a flow rate of 0.4 mL/min and 0.3 mM ammonium fluoride in H_2_O as eluent A and ACN as eluent B. Details are reported in Table S6. A QTrap 6500+ mass spectrometer equipped with an electrospray ionization (ESI) source (Sciex) was operated using a previously published multiple reaction monitoring (MRM) methods with fast polarity switching.^6^ Full method parameters including MRM transitions and ESI parameters are provided in the SI.

#### Nontargeted HRMS(/MS)

An Orbitrap Exploris 480 mass spectrometer (Thermo Scientific) was used for HRMS(/MS) measurements. Samples were analyzed using full scan mode and AcquireX was used to generate fragment spectra using a mass resolution of 120,000 at *m*/*z* 200, with full method parameters reported in the SI.

### Data Analysis

#### Quality Control

Several types of QC samples were used to ensure reliable results. A systems suitability test sample (SST) was measured before and after each analytical sequence to ensure favorable instrument performance (see SI and Table S7). In addition, a multianalyte reference standard mixture consisting of 94 highly diverse chemicals in pure solvent was injected. Solvent blanks were used to monitor instrumental background and carryover and process blanks were used to monitor background concentrations/contaminations.

#### Targeted Data Analysis

RE and SSE values were evaluated for the set of 94 analytes^6^ using standards in neat solvent, pooled urine, and pooled plasma. Peak integration was carried out using Multiquant (v3.0.2, Sciex) and Skyline (v21.2.2.536, McCoss Lab).^30^ Calculation of RE and SSE was performed using Microsoft Office 365. Graphics were created by Origin 2021b (v9.8; OriginLab Corporation).

#### Nontargeted Data Analysis

Raw data files were analyzed using MSDIAL (v5.2).^31^ Full data processing settings including data extraction, peak picking, compound identification, and alignment are reported in the SI. To evaluate the qualitative analyte coverage for NTA and suspect screening, MassBank of North America (MoNA, https://mona.fiehnlab.ucdavis.edu/) was used as a spectral library. Annotations were then classified based on the level of confidence scheme according to Schymanski et al.^32^ using confidence levels 1 (confirmed by authentic standards), level 2 (confirmed by spectral library match), and level 3 (confirmed by spectral library match, but with isomeric structures). Additional data analysis and visualization was performed using Microsoft Office 365, Origin 2021b, MetaboAnalyst (v6.0), and Inkscape (version 1.2.2; Inkscape).

## Results and Discussion

### SPE Sorbent Selection

Analyte loss was compared for both SPE sorbents and HLB and PSA+C18 showed comparable performance (see Figure S1). “Good” performance (analyte loss <10% during loading and washing) was achieved for 90% of all analytes, whereas “good” RE was achieved for 59% of all analytes using HLB compared to 39% using the mixed-mode sorbent PSA+C18.

Analyte loss >5% was observed for six analytes with LogP values between 1.7 and 3.2 when using the PSA+C18 for SPE, whereas analyte loss >5% was only observed for a single compound when using the HLB sorbent (see Figure S2). Most analytes showed sufficient RE (50–150%) using either of the two sorbents but RE <50% were observed for 15 analytes using the mixed-mode sorbent, whereas only six analytes with RE <50% were observed using the HLB sorbent. Consequently, the HLB was selected as the sorbent material for further workflow optimization.

### Buffer Selection for Sample before Loading

Subsequently, the buffer system used for sample loading was optimized. As shown in Figure S3, >95% of all analytes showed analyte losses <10% after diluting samples with PBS buffer. This fraction dropped to 80% and 87% of the analytes when using NH_4_Ac and H_2_O as dilution solvents. Consequently, PBS was chosen as the sample buffer for further process optimization.

### Washing, Elution, and Reconstitution Optimization

Thereafter, washing, elution and reconstitution steps were optimized. Matrix effects were investigated in terms of SSE (%) and results for the two tested washing protocols were largely comparable (Figure S4). However, using two times 1 mL of H_2_O improved SSE by approximately 5% for most analytes, especially for those with RT between 1.0 and 7.0 min (Figure S5). Consequently, two washing cycles using 1 mL H_2_O each were used in the final protocol.

**Figure 1:**
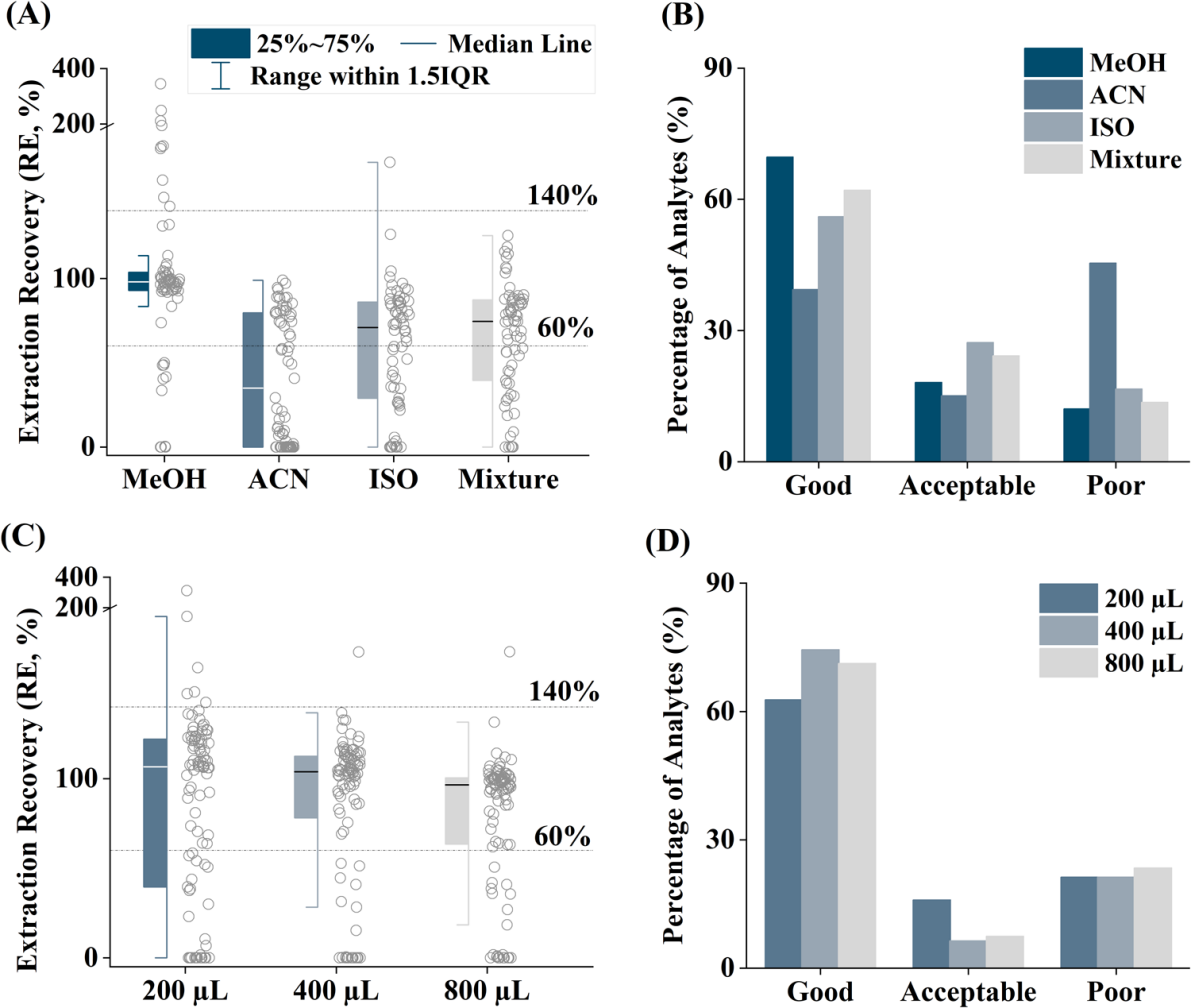
Extraction recovery (RE, %) for 94 analytes in pooled urine using HLB cartridges with different elution conditions (A) and classification of the performance into three categories (B) using methanol (MeOH), acetonitrile (ACN), isopropanol (ISO), and a mixture of ISO/MeOH/ACN (1/1/1, v/v/v). Panels (C) and (D) show results for the optimization of the elution volume for MeOH (200 μL, 400 μL, and 800 μL). The following classification was used in panels (B) and (D): “Good”: RE 60−140%, “Acceptable”: RE 20–60% or 140–180%, “Poor”: RE <20% or RE >180%.

The use of acidic MeOH solutions (3% FA) as elution solvent resulted in lower RE (<50% for most analytes), whereas better RE (50–150% for most analytes) was achieved using pure MeOH or basic MeOH (3% NH_3_) for elution. Six nonpolar compounds (LogP, 4.0–5.5) had surprisingly high RE values >150% using the basic MeOH solution while only a single compound had RE >150% using pure MeOH (Figure S2 (F)). Thus, pure MeOH was selected as the elution solvent for further optimization. In addition, it should be noted that basic solvents with high ammonia concentrations may cause issues including unstable RTs and potential damage to both the UPLC column sorbent and the UPLC system itself.^33^ Different elution solvents (MeOH, ACN and ISO) were compared and elution volumes were optimized. “Good” REs were achieved for approximately 70% using MeOH for elution, whereas this reduced to 39–62% when ACN, ISO or a 1:1:1 mixture of all solvents was used (Figure 1).

Solvent volumes were optimized for MeOH and 200 μL, 400 μL, and 800 μL were tested for elution (see Figure 1C,D). Approximately 70% of compounds achieved “good” REs when using 400 μL or 800 μL of MeOH for elution, whereas this fraction dropped to 63% when eluting with 200 μL MeOH. The smaller solvent volumes (200 μL) resulted in inefficient elution for around 10% of all analytes and 400 μL (using 2 times 200 μL) was selected for further method optimization, as it represented a well-balanced compromise between good RE and low solvent consumption. Regardless of the elution conditions, certain highly polar compounds in pooled urine were poorly retained on the SPE cartridges. These compounds were primarily low-mass organic acids such as 5-hydroxymethyl-2-furanoic acid, dibromoacetic acid, dichloroacetic acid, bromoacetic acid, and p-hydroxybenzoic acid and were also only weakly retained by the reversed-phase (RP-)LC column with the applied method (i.e., RT <1 min).

The effect of drying the raw extracts followed by reconstitution was investigated (see Figure S6), and diluting the raw extracts resulted in improved recoveries, indicating the need to consider the optimization of reconstitution steps during method development. Approximately 6% of analytes with “good” REs using the original protocol (i.e., SPE followed by dilution), fell into the “acceptable” category after drying and reconstitution of the extracts. Potential causes include short vortexing, the absence of sonication steps, or the selection of the resuspension solvent containing 50% MeOH in H_2_O (1/1, v/v). For example, the RE for 2-*tert*-butylphenol decreased significantly from 99.7% with dilution to 23% after drying and reconstitution. Furthermore, the study found an increased background signal for certain plastics related exposures including bisphenol F and S, when using drying and reconstitution. Consequently, a direct dilution step is clearly favorable in terms of simplicity and speed, which are both required for high-throughput applications.

### Transfer from Cartridges to 96-Well Plate Format

For maximizing sample throughput, the SPE protocol was transferred to 96-well plates (plate-based SPE) and performance was compared to the initially optimized cartridge-based SPE protocol. In total, 72% of all analytes fulfilled the criteria for “good” RE when using the cartridge-based protocol for extracting urine or plasma samples, and 86% and 90% of the analytes were classified as “good” in terms of SSE. When using 96-well plates, 71% and 60% of the analytes achieved “good” REs while 76% and 78% of all analytes obtained “good” SSE in urine and plasma, respectively. Most analytes with “good” REs using the cartridge-based workflow were also categorized as “good” using the 96-well plate workflow for the extraction of urine, whereas the performance of the 96-well plate method was reduced for 12% of the analytes in plasma. Similarly, the cartridge-based protocol outperformed the plate-based SPE method in 10% of the cases in terms of matrix-induced SSE (see Figure S7).

**Figure 2:**
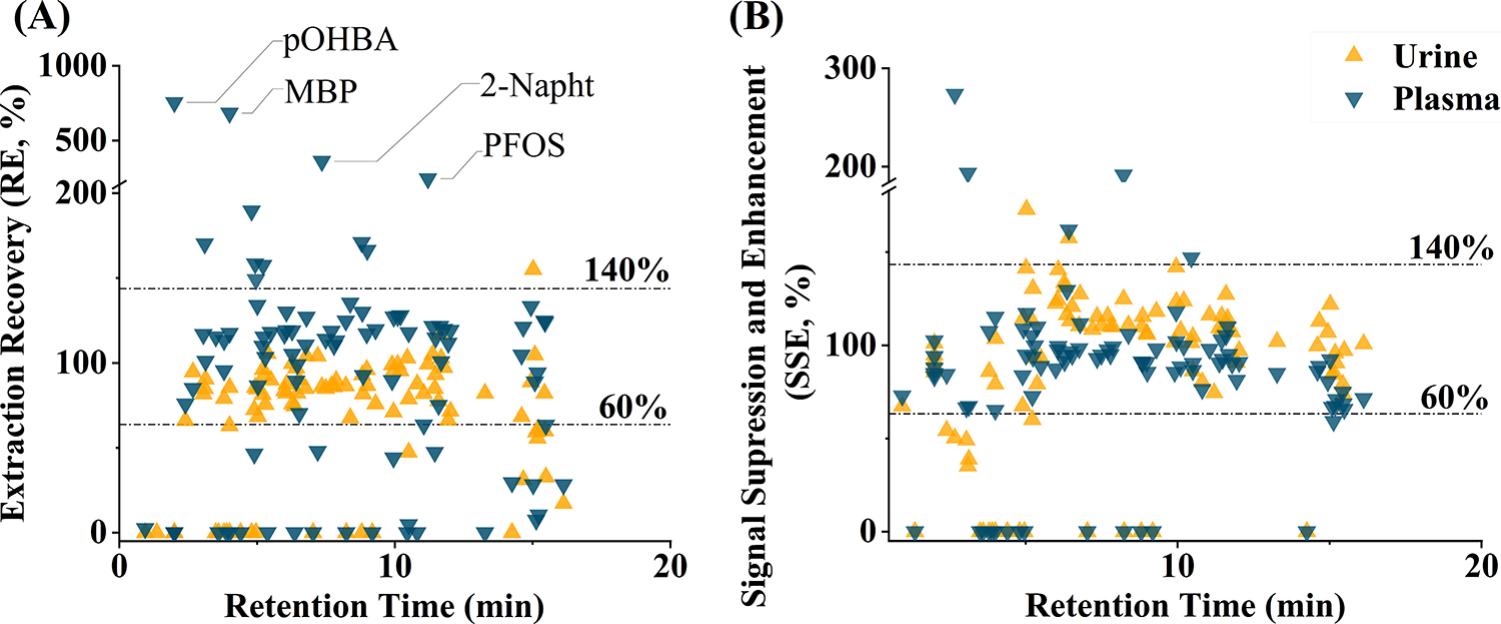
Extraction recoveries (A) and signal suppression and enhancement (SSE, %) (B) for 94 analytes in urine (yellow) and plasma (blue) using the optimized protocol based on SPE 96-well plate format. pOHBA, p-hydroxybenzoic acid; MBP, monobutyl phthalate; 2-Napht, 2-naphthol; PFOS, perfluorooctanesulfonic acid.

**Figure 3:**
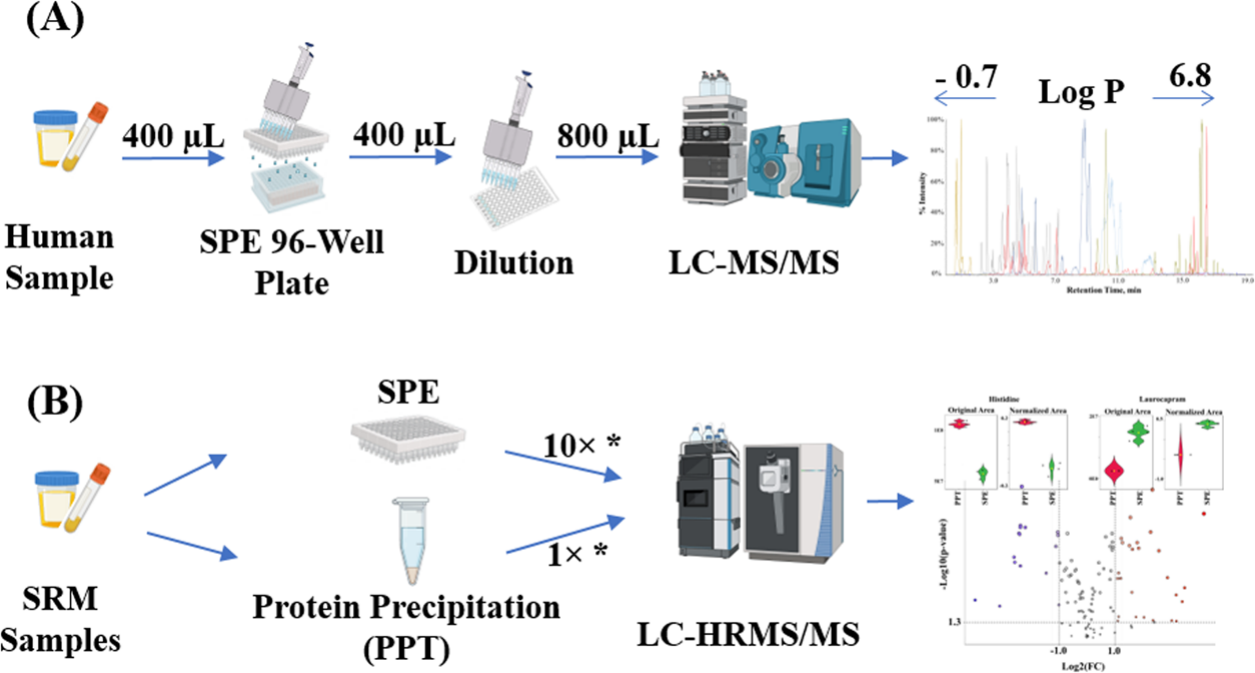
(A) SPE workflow for targeted analysis. (B) Illustration of the feasibility test of the established SPE method for nontargeted analysis (NTA). * The SPE protocol in 96-well plate format is approximately 10× faster than a state-of-the-art protein precipitation workflow when calculated for 1000 samples. The −0.7 to 6.8 logP range indicates the workflow’s ability to cover chemicals with varying polarities.

In total, around 25% of the analytes were poorly extracted from plasma and 20% from urine using the plate-based protocol. The observed discrepancies might be explained by differences in the manufacturing process, including sorbent packing, or differences in sample handling when working with 96-well plates. In addition to weakly retained compounds (low-mass acids, see above), additional compounds in plasma, such as p-hydroxybenzoic acid (pOHBA), monobutyl phthalate (MBP), 2-naphthol (2-Napht) or perfluorooctanesulfonic acid (PFOS), were reported with “poor” RE. This was due to their background levels being 2 to 10 times higher than the spiked levels. In general, RE and SSE were only slightly better when using cartridges compared to 96-well plates (see Figure 2). Despite the slightly reduced performance in selected examples, the application of 96-well plates still holds promise for large-scale applications due to its comparable performance to the cartridges in combination with notable improvement in sample throughput, and handling. A schematic representation of the SPE workflow based on 96-well plates is presented in Figure 3 and more details are given in Table S8. To assess the full potential for high-throughput applications, the total analysis time of the 96-well plate-based SPE workflow was predicted for the analysis of 1000 samples, and time estimates were compared to our routinely used PPT protocol.^29^ The new plate-based SPE protocol allows extracting 1000 biological samples within 3 days, whereas the same amount of samples would require 29 days using a routinely used PPT workflow as shown in Table S9.

### Assessing the Potential of SPE for NTA and Suspect Screening

While systematic optimization and characterization of sample preparation methods for targeted (multianalyte) assays can be performed easily, full-performance comparison for nontargeted applications is less straightforward. We aimed to characterize the applicability of the developed SPE method for NTA and suspect screening, by comparing performance to a well-established protein precipitation method (PPT). Thus, extracts obtained with PPT and 96-well plate-based SPE were analyzed using LC-HRMS(/MS) (see Figure 3B), and results were compared on the feature level. Extracts of NIST SRM 1950 (plasma) and SRM 3672 (urine) were analyzed on an LC-HRMS instrument. Peak picking, feature alignment and compound annotation were performed using MSDIAL together with reference mass spectra from MassBank of North America (MoNA). Relative intensities were determined for annotated features as the area under curve (AUC), and results of the PPT and SPE workflows were compared for annotated and identified features. Values of fold change (FC >2) and significance (*p*-value <0.05) were analyzed using MetaboAnalyst.^34^ Approximately 50% of the annotated features detected in SRM 1950 (plasma), showed significantly higher AUCs (FC >2, *p*-value <0.05) when using the PPT workflow (see Table S10). Yet, SPE extraction resulted in significantly higher signal intensities for 13% of the features, and 36% of the features were not significantly affected by the choice of sample preparation. For SRM 3672 (urine), we observed significantly higher AUCs using PPT in 32% of cases, whereas 10% were significantly enhanced using SPE, and 58% of annotated features were not significantly different (Table S11).

**Figure 4:**
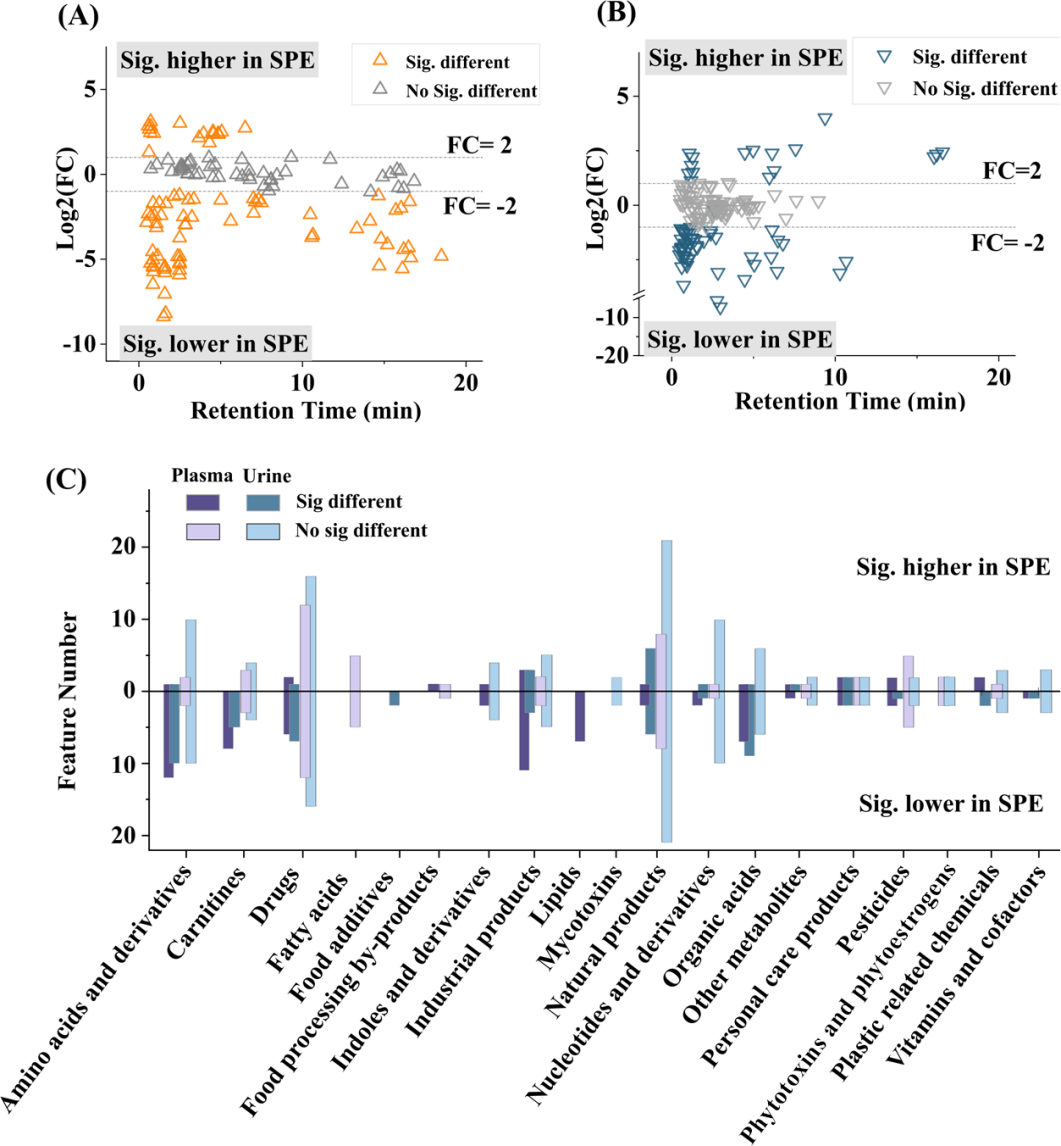
Evaluation of SPE performance compared to protein precipitation for nontargeted analysis (NTA). Comparison of peak areas of annotated features (confidence levels 1–3) in SRM 1950 plasma (A) and SRM 3672 urine (B) as a function of retention time as log2 fold change of the peak area, and summary of the results based on the compound class (C).

Figure 4A,B shows results of the comparison of relative feature intensities as fold changes of AUC as a function of the RT, whereas results are summarized on the compound class level in Figure 4C. For SRM 1950 (plasma), higher relative signal intensities in PPT extracts were observed for polar compounds, mainly amino acids and organic acids with RT <3 min, which is well in line with the results from the targeted assay. Additionally, several nonpolar features, mainly lipids with RT >10 min were higher in the PPT extracts. Similar results were observed for SRM 3672 urine, with polar compounds including amino acids and derivatives or organic acids with RT <3 min showing higher AUCs in PPT extracts compared to SPE. However, some key low-abundance biomarkers were observed with significantly higher AUCs using SPE, like nicotine in urine (SRM 3672) and cocaine in plasma (SRM 1950, see Figure S8). Interestingly, no significant differences between PPT and SPE were observed for major metabolites of nicotine and cocaine such as benzoylecgonine^35^ for cocaine, and hydroxycotinine and cotinine for nicotine.

In conclusion, the PPT workflow offers wider coverage of annotated features, but comparable results were obtained for many annotated compounds. In urine, the results were comparable for 58% of annotated features detected in extracts using both methods. However, PPT extraction provided broader feature coverage for the analysis of plasma samples, particularly endogenous polar metabolites, whereas SPE extraction improved the detection of low-abundance exogenous compounds at the cost of losing several polar metabolites.

## Limitations

While the established workflow is effective for most analytes in targeted multiclass analysis of human samples and performs comparably well for a relative majority of analytes in NTA, several limitations remain. The analytes selected for method optimization were highly diverse and yet still represent only a limited subset of the known chemical exposome. Although analytes were selected with a special emphasis on covering a wide range of physicochemical properties, coverage needs to be further expanded for exposome-scale work. A broader selection of compounds and wider chemical coverage would be needed for a truly holistic representation of the exposome in future work. Moreover, certain analytes, especially polar acidic compounds, are partly lost during the SPE process due to weak retention by the sorbent, which was expected. Although different workflows could address these specific shortcomings by targeting particular sets of analytes, conducting multiple measurements to gather sufficient information is time-consuming. Therefore, achieving favorable performance in terms of comprehensive analyte coverage, efficient cleanup, and minimal time consumption simultaneously is not always feasible and not always required.^36^

## Conclusions and Outlook

## Supporting Information Available

Methods and the results; Figures S1–S8; and references (PDF)
Tables S1–S11 (XLSX)

## References

[ref1] Krausová M.; Braun D.; Buerki-Thurnherr T.; Gundacker C.; Schernhammer E.; Wisgrill L.; Warth B. Understanding the Chemical Exposome During Fetal Development and Early Childhood: A Review. Ann. Rev. Pharmacol. Toxicol. 2023, 63 (1), 517 10.1146/annurev-pharmtox-051922-113350. 36202091

[ref2] Walker D. I.; Juran B. D.; Cheung A. C.; Schlicht E. M.; Liang Y.; Niedzwiecki M.; LaRusso N. F.; Gores G. J.; Jones D. P.; Miller G. W.; Lazaridis K. N. High-Resolution Exposomics and Metabolomics Reveals Specific Associations in Cholestatic Liver Diseases. Hepatol. Commun. 2022, 6 (5), 965–979. 10.1002/hep4.1871. 34825528 PMC9035559

[ref3] Oesterle I.; Ayeni K. I.; Ezekiel C. N.; Berry D.; Rompel A.; Warth B. Insights into the early-life chemical exposome of Nigerian infants and potential correlations with the developing gut microbiome. Environ. Int. 2024, 188, 108766 10.1016/j.envint.2024.108766. 38801800

[ref4] Wild C. P. Complementing the genome with an ″exposome″: the outstanding challenge of environmental exposure measurement in molecular epidemiology. Cancer Epidemiol., Biomarkers Prev. 2005, 14 (8), 1847–1850. 10.1158/1055-9965.EPI-05-0456. 16103423

[ref5] Svarcova A.; Lankova D.; Gramblicka T.; Stupak M.; Hajslova J.; Pulkrabova J. Integration of five groups of POPs into one multi-analyte method for human blood serum analysis: An innovative approach within biomonitoring studies. Sci. Total Environ. 2019, 667, 701–709. 10.1016/j.scitotenv.2019.02.336. 30849610

[ref6] Jamnik T.; Flasch M.; Braun D.; Fareed Y.; Wasinger D.; Seki D.; Berry D.; Berger A.; Wisgrill L.; Warth B. Next-generation biomonitoring of the early-life chemical exposome in neonatal and infant development. Nat. Commun. 2022, 13 (1), 2653 10.1038/s41467-022-30204-y. 35550507 PMC9098442

[ref7] Pourchet M.; Debrauwer L.; Klanova J.; Price E. J.; Covaci A.; Caballero-Casero N.; Oberacher H.; Lamoree M.; Damont A.; Fenaille F.; et al. Suspect and non-targeted screening of chemicals of emerging concern for human biomonitoring, environmental health studies and support to risk assessment: From promises to challenges and harmonisation issues. Environ. Int. 2020, 139, 105545 10.1016/j.envint.2020.105545. 32361063

[ref8] Musatadi M.; Caballero C.; Mijangos L.; Prieto A.; Olivares M.; Zuloaga O. From target analysis to suspect and non-target screening of endocrine-disrupting compounds in human urine. Anal Bioanal Chem. 2022, 414 (23), 6855–6869. 10.1007/s00216-022-04250-w. 35904524 PMC9436830

[ref9] Oesterle I.; Pristner M.; Berger S.; Wang M.; Verri Hernandes V.; Rompel A.; Warth B. Exposomic biomonitoring of polyphenols by non-targeted analysis and suspect screening. Anal. Chem. 2023, 95 (28), 10686–10694. 10.1021/acs.analchem.3c01393. 37409760 PMC10357401

[ref10] Wang A.; Gerona R. R.; Schwartz J. M.; Lin T.; Sirota M.; Morello-Frosch R.; Woodruff T. J. A Suspect Screening Method for Characterizing Multiple Chemical Exposures among a Demographically Diverse Population of Pregnant Women in San Francisco. Environ. Health Perspect 2018, 126 (7), 077009 10.1289/EHP2920. 30044231 PMC6108847

[ref11] Dennis K. K.; Marder E.; Balshaw D. M.; Cui Y.; Lynes M. A.; Patti G. J.; Rappaport S. M.; Shaughnessy D. T.; Vrijheid M.; Barr D. B. Biomonitoring in the Era of the Exposome. Environ. Health Perspect 2017, 125 (4), 502–510. 10.1289/EHP474. 27385067 PMC5381997

[ref12] Cano-Sancho G.; Alexandre-Gouabau M. C.; Moyon T.; Royer A. L.; Guitton Y.; Billard H.; Darmaun D.; Roze J. C.; Boquien C. Y.; Le Bizec B.; Antignac J. P. Simultaneous exploration of nutrients and pollutants in human milk and their impact on preterm infant growth: An integrative cross-platform approach. Environ. Res. 2020, 182, 109018 10.1016/j.envres.2019.109018. 31863943

[ref13] Chen X.; Wu X.; Luan T.; Jiang R.; Ouyang G. Sample preparation and instrumental methods for illicit drugs in environmental and biological samples: A review. J. Chromatogr A 2021, 1640, 461961 10.1016/j.chroma.2021.461961. 33582515

[ref14] Preindl K.; Braun D.; Aichinger G.; Sieri S.; Fang M.; Marko D.; Warth B. A Generic Liquid Chromatography-Tandem Mass Spectrometry Exposome Method for the Determination of Xenoestrogens in Biological Matrices. Anal. Chem. 2019, 91 (17), 11334–11342. 10.1021/acs.analchem.9b02446. 31398002

[ref15] Nováková L.; Vlckova H. A review of current trends and advances in modern bio-analytical methods: chromatography and sample preparation. Anal. Chim. Acta 2009, 656 (1–2), 8–35. 10.1016/j.aca.2009.10.004. 19932811

[ref16] Ötles S.; Kartal C. Solid-Phase Extraction (SPE): Principles and Applications in Food Samples. Acta Sci. Pol Technol. Aliment 2016, 15 (1), 5–15. 10.17306/J.AFS.2016.1.1. 28071034

[ref17] Warth B.; Spangler S.; Fang M.; Johnson C. H.; Forsberg E. M.; Granados A.; Martin R. L.; Domingo-Almenara X.; Huan T.; Rinehart D.; et al. Exposome-Scale Investigations Guided by Global Metabolomics, Pathway Analysis, and Cognitive Computing. Anal. Chem. 2017, 89 (21), 11505–11513. 10.1021/acs.analchem.7b02759. 28945073

[ref18] Gu Y.; Peach J. T.; Warth B. Sample preparation strategies for mass spectrometry analysis in human exposome research: Current status and future perspectives. TrAC, Trends Anal. Chem. 2023, 166, 117151 10.1016/j.trac.2023.117151.

[ref19] Jagani R.; Pulivarthi D.; Patel D.; Wright R. J.; Wright R. O.; Arora M.; Wolff M. S.; Andra S. S. Validated single urinary assay designed for exposomic multi-class biomarkers of common environmental exposures. Anal. Bioanal. Chem. 2022, 414 (19), 5943–5966. 10.1007/s00216-022-04159-4. 35754089 PMC9326253

[ref20] Šarkanj B.; Ezekiel C. N.; Turner P. C.; Abia W. A.; Rychlik M.; Krska R.; Sulyok M.; Warth B. Ultra-sensitive, stable isotope assisted quantification of multiple urinary mycotoxin exposure biomarkers. Anal. Chim. Acta 2018, 1019, 84–92. 10.1016/j.aca.2018.02.036. 29625687

[ref21] Palát J.; Kukučka P.; Codling G. P.; Price E. J.; Janků P.; Klánová J. Application of 96-well plate SPE method for analysis of persistent organic pollutants in low volume blood serum samples. Chemosphere 2022, 287, 132300 10.1016/j.chemosphere.2021.132300. 34563784

[ref22] Chen M.; Koekkoek J.; Lamoree M. Organophosphate ester metabolites in human breast milk determined by online solid phase extraction coupled to high pressure liquid chromatography tandem mass spectrometry. Environ. Int. 2022, 159, 107049 10.1016/j.envint.2021.107049. 34952374

[ref23] Papavasileiou A. V.; Trachioti M. G.; Hrbac J.; Prodromidis M. I. Simultaneous determination of guanine and adenine in human saliva with graphite sparked screen-printed electrodes. Talanta 2022, 239, 123119 10.1016/j.talanta.2021.123119. 34864536

[ref24] Jia W.; Liu H.; Ma Y.; Huang G.; Liu Y.; Zhao B.; Xie D.; Huang K.; Wang R. Reproducibility in nontarget screening (NTS) of environmental emerging contaminants: Assessing different HLB SPE cartridges and instruments. Sci. Total Environ. 2024, 912, 168971 10.1016/j.scitotenv.2023.168971. 38042181

[ref25] Matuszewski B. K.; Constanzer M.; Chavez-Eng C. Strategies for the assessment of matrix effect in quantitative bioanalytical methods based on HPLC– MS/MS. Anal. Chem. 2003, 75 (13), 3019–3030. 10.1021/ac020361s. 12964746

[ref26] Braun D.; Schernhammer E.; Marko D.; Warth B. Longitudinal assessment of mycotoxin co-exposures in exclusively breastfed infants. Environ. Int. 2020, 142, 105845 10.1016/j.envint.2020.105845. 32563012

[ref27] Turković L.; Mutavdžić Pavlović D.; Mlinarić Z.; Skenderović A.; Silovski T.; Sertić M. Optimisation of Solid-Phase Extraction and LC-MS/MS Analysis of Six Breast Cancer Drugs in Patient Plasma Samples. Pharmaceuticals 2023, 16 (10), 1445 10.3390/ph16101445. 37895916 PMC10610126

[ref28] Oosterwegel M. J.; Ibi D.; Portengen Lt.; Probst-Hensch N.; Tarallo S.; Naccarati A.; Imboden M.; Jeong A.; Robinot N.; Scalbert A.; et al. Variability of the Human Serum Metabolome over 3 Months in the EXPOsOMICS Personal Exposure Monitoring Study. Environ. Sci. Technol. 2023, 57 (34), 12752–12759. 10.1021/acs.est.3c03233. 37582220 PMC10469440

[ref29] Flasch M.; Koellensperger G.; Warth B. Comparing the sensitivity of a low-and a high-resolution mass spectrometry approach for xenobiotic trace analysis: An exposome-type case study. Anal. Chim. Acta 2023, 1279, 341740 10.1016/j.aca.2023.341740. 37827628

[ref30] Adams K. J.; Pratt B.; Bose N.; Dubois L. G.; St John-Williams L.; Perrott K. M.; Ky K.; Kapahi P.; Sharma V.; MacCoss M. J.; et al. Skyline for small molecules: a unifying software package for quantitative metabolomics. J. Proteome Res. 2020, 19 (4), 1447–1458. 10.1021/acs.jproteome.9b00640. 31984744 PMC7127945

[ref31] Tsugawa H.; Cajka T.; Kind T.; Ma Y.; Higgins B.; Ikeda K.; Kanazawa M.; VanderGheynst J.; Fiehn O.; Arita M. MS-DIAL: data-independent MS/MS deconvolution for comprehensive metabolome analysis. Nat. Methods 2015, 12 (6), 523–526. 10.1038/nmeth.3393. 25938372 PMC4449330

[ref32] Schymanski E. L.; Jeon J.; Gulde R.; Fenner K.; Ruff M.; Singer H. P.; Hollender J. Identifying small molecules via high resolution mass spectrometry: communicating confidence. Environm. Sci. Technol. 2014, 48, 2097 10.1021/es5002105. 24476540

[ref33] Kirkland J. J.; Van Straten M.; Claessens H. High pH mobile phase effects on silica-based reversed-phase high-performance liquid chromatographic columns. J. Chromatogr. A 1995, 691 (1–2), 3–19. 10.1016/0021-9673(94)00631-I. 9098970

[ref34] Pang Z.; Lu Y.; Zhou G.; Hui F.; Xu L.; Viau C.; Spigelman A. F.; MacDonald P. E.; Wishart D. S.; Li S.; Xia J. MetaboAnalyst 6.0: towards a unified platform for metabolomics data processing, analysis and interpretation. Nucleic Acids Res. 2024, 52, gkae253 10.1093/nar/gkae253. PMC11223798 38587201

[ref35] Souza A.; Ntai I.; Tautenhahn R. Accelerated unknown compound annotation with confidence: from spectra to structure in untargeted metabolomics experiments Thermo Fisher Scientific Application Note 65362, 2018.

[ref36] Miller G. W. Exposomics: Perfection Not Required; Exposome, Oxford University Press, 2024; Vol. 4, p osae006. 39372501 10.1093/exposome/osae006 PMC11450953

