# Supplementary material for: High-Throughput Solid Phase Extraction for Targeted and Nontargeted Exposomics
Source: Anal Chem. 2025 Mar 13;97(11):6075–82. doi: 10.1021/acs.analchem.4c06177 (PMC11948174; doi:10.1021/acs.analchem.4c06177)
Supplement: Supplementary file 1 — ac4c06177_si_001.pdf [file ac4c06177_si_001.pdf]

Supplementary Information

High-Throughput Solid Phase Extraction for Targeted and Non-Targeted Exposomics

Yunyun Gu[a,b], Max L. Feuerstein[a,c], Benedikt Warth[a,b,c,*]

[a]University of Vienna, Faculty of Chemistry, Department of Food Chemistry and Toxicology, 1090, Vienna, Austria
[b]University of Vienna, Vienna Doctoral School of Chemistry, Währinger Straße 42, 1090, Vienna, Austria
[c]Exposome Austria, Research Infrastructure and National EIRENE Node, Austria

*Corresponding author: Benedikt Warth, benedikt.warth@univie.ac.at, +43-1-4277-70806.

Table of Contents

Materials.....2

Mixture Standards.....2

Preparation of In-house Mix-mode SPE Cartridges (PSA+C18). 2

Spiking Process. 2

Sample preparation with SPE. 2

Instrumental Analysis. 3

References. 10

Materials.

Reference standards, stocks, solutions, reagents, and solvents were used according to our previous research[1]. All chemical stocks and solutions were stored at -20°C. In this study, 81 xenoestrogens and 13 endogenous estrogens were included in the targeted analysis (**Table S1-2),** and a total of 234 standards were used for compound conformation during the nontargeted analysis and suspect screening (**Table S2)**. All chemical stocks and solutions were stored at -20 °C. For solid phase extraction (SPE), Oasis PRiME HLB cartridges (30 mg, 1cc) and 96-well plates (30 mg, 2 mL) were purchased from Waters Corporation (Vienna, Austria). Methanol (MeOH), acetonitrile (ACN), and water (H_2O) used in this study are LC-MS grade. Suppliers and CAS numbers of used standards are provided in **Table S2**.

Standard mixtures.

A standard mixture of 94 chemicals was spiked into pure solvent, pooled urine or pooled plasma as a sample matrix for optimizing the results for targeted analysis. To speed up the data analysis process, 66 analytes were carefully selected as a representative subset for preliminary optimization of SPE steps, including the selection of SPE sorbents and sample buffer. All 94 standards were used to optimize and fine-tune the final SPE method. To test the applicability of the developed SPE

workflow for NTA, NIST SRM 1950 (plasma) and SRM 3672 (urine) were analyzed using LC-HRMS(/MS). To complement annotations based on spectral library search, a standard mix containing 234 compounds was spiked into both sample matrices to improve coverage and level of confidence of compound annotation ("level 1": identified based using authentic standards). The standard mix used for system suitability tests (SST) includes caffeine, phenylalanine, roxithromycin, zearalenone, bisphenol A, genistein, and fipronil. Details are reported in **Table S8**.

Preparation of In-house Mix-mode SPE Cartridges (PSA+C18).

SPE sorbents, including C18 and primary second amine (PSA), were purchased from Sigma-Aldrich (Merck, Germany). Clean empty PP cartridges (1 mL) with 10 µm PE-filter elements were purchased from BEKOlut GmbH & Co.KG (Bruchmühlbach-Miesau, Germany). The two types of sorbents were first thoroughly mixed using equal weights. Then, 30 mg of the mixed sorbent were tightly packed into each clean cartridge, with two filter elements placed at the bottom and top of the sorbent layer.

Spiking Process.

The term "prespiked sample" refers to samples spiked with a mixture of standards before SPE extraction and the term "postspiked sample" indicates spiking after the extraction. Pairs of "prespiked" and "postspiked" samples were prepared to evaluate results of all individual optimization steps during the SPE method development. Four replicates were used to ensure repeatability of the results. Spiked concentrations are provided in **Table S3**. Analyte loss during loading and washing steps was used to describe the fraction of weakly retained analyte during SPE loading and washing. Therefore, effluents of loading and washing steps were collected, dried, reconstituted, and analyzed using LC-MS/MS. The analyte loss (%) was determined as the ratio of peak areas of effluents from the "prespiked" sample to peak areas observed in the "postspiked" effluents. Extraction recovery (RE, %) was determined as ratios of peak areas of pairs of "prespiked" and "postspiked" samples. Matrix effects were evaluated in terms of signal suppression and enhancement (SSE, %) and were calculated for urine and plasma as the ratio of peak areas for "postspiked" samples, to standards in pure water (spiked after going through the full SPE process)[2]. Peak areas of compounds determined for pure water, pooled urine, and pooled plasma were used to subtract the method background. Additionally, "postspiked" SRM samples were used in nontargeted analysis to improve the annotation confidence ("level 1": identified based on authentic chemical standards).

Sample preparation with SPE.

Overview for Development and Optimization of SPE Workflow.

As indicated in **Table S8**, the sample pretreatment of the SPE workflow was developed and optimized in five steps, including selection of SPE sorbents, selection of sample buffer, SPE elution optimization, and SPE washing and reconstitution. As indicated in **Table S8**, HLB was selected as the SPE sorbent, 400 µL of PBS was chosen as the sample buffer, 400 µL of human urine or plasma was diluted with PBS, 2 mL of H_2O was chosen as the SPE washing solvent, 400 µL of MeOH was selected for the SPE elution, and direct dilution of 400 µL of the SPE extract with 400 µL of H_2O was selected as the final constitution before LC-MS/MS analysis.

SPE workflow.

The final and optimized SPE workflow was as follows: For sample extraction and sample clean-up, either SPE cartridges or 96-well plates were used. SPE sorbents were conditioned with 1 mL of methanol (MeOH), followed by 1 mL of water (H_2O). Subsequently, 400 µL of urine or plasma sample were mixed with an equal volume (400 µL) of phosphate-buffered saline (PBS, pH 7.4). The resulting 800 µL solution was loaded onto the SPE sorbents. The sorbents were then washed twice with 1 mL of H_2O each. Following the washing steps, the sorbents were dried by applying a negative vacuum of -30 kPa using a 96-well plate manifold for 5 minutes. Analytes were then eluted using two times 200 µL of MeOH (400 µL in total). Throughout the procedure, the drop rate was strictly controlled, maintained at less than 1 drop per second by SPE cartridge manifold, or allowed to proceed solely under gravity when using SPE in 96-well plates, in order to obtain stable and consistent recovery values. After the elution, the mixture was diluted by adding 400 µL of H_2O, to yield a 1/1 (v/v) composition of MeOH/H_2O and samples were vortexed before LC-MS/MS analysis.

From Targeted to Non-targeted Analysis.

To test the applicability for NTA, two sample pretreatment methods were compared for plasma SRM1950 and urine SRM3672 samples. Therefore, we analyzed SRM samples, together with solvent standards, and postspiked SRM samples spiked with concentrations given in **Table S4**. A slightly adjusted protein precipitation (PPT) method was used according to our previous method[1,3]. Briefly, a volume of 100 µL sample was extracted with 400 µL of the extraction solvent (ACN/MeOH, 1/1, v/v) by sonication (10 min, 4 °C). After precipitating proteins in a freeze-out step (2 h, - 20 °C), the samples were evaporated and reconstituted in 100 µL of 10% ACN in H_2O (ACN/ H_2O, 10/90, v/v). For SPE process, to get comparable results, the sample volume for SPE method were reduced from 400 µL to 100 µL by drying sample extracts followed by reconstitution. Briefly, 100 µL of sample were mixed with 100 µL of PBS and all remaining steps were the same as in the previous description of the SPE workflow. The resulting 400 µL of SPE extracts were dried by a SpeedVac and reconstituted with 100 µL of 10% ACN in H_2O (ACN/ H_2O, 10/90, v/v).

Instrumental Analysis.

UPLC-MS/MS Analysis.

The applied chromatographic separation was based on our previous method[1]. Briefly, an Acquity HSS T3 reversed-phase column (1.8 µm, 2.1× 100 mm, Waters) and a VanGuard pre-column (1.8 µm) (Waters) were installed on a 1290 Infinity II LC system (Agilent). Eluent A was 0.3 mM of ammonium fluoride in LC-MS grade water and eluent B was ACN. A volume of 5 µL of sample was injected into the system at a 0.4 mL/min flow rate. The column compartment and autosampler were maintained at 40°C and 7°C, respectively. The used LC-gradient was the following: 0.0 - 1.0 min, 5% B; rise to 18% B until 1.8 min and to 35% B until 4.2 min; rise to 48% B until 13.0 min and to 90% B until 14.0 min; rise to 98% until 15.8 min and flush with 98% B from 15.8 min to 17.6 min; re-equilibrate with 5% B from 17.7 min to 20.0 min. The gradient table is described in the **Table S6**. A QTrap 6500+ mass spectrometer equipped with an electrospray ionization (ESI) source (Sciex) was coupled to the UPLC system. Fast polarity switching in multiple reaction monitoring (MRM) mode was utilized. The MRM transitions in this study were according to our previous study[1].

UPLC-HRMS(/MS) for Non-Targeted Analysis (NTA).

A Thermo Fisher Scientific Orbitrap Exploris™ 480 mass spectrometer coupled to a Vanquish UHPLC system was used for the HRMS(/MS) measurements using a H-ESI (heated electrospray ionization) source. To ensure optimal comparability, the experimental setup was closely related to the setup used for low-resolution LC-MS/MS measurements (see above), with identical column, eluents, gradients, and other LC parameters. The injection volume was set to 3 µL. Full Scan MS^1 spectra were recorded over a mass range of 60-900 m/z with Orbitrap resolution set at 90,000 and the RF (radio frequency) lens set to 50%. AGC target (automatic gain control) was set to “standard” and the maximum injection time was set to “auto”. MS^2 data was acquired using AcquireX for each polarity separately and exclusion lists and inclusion lists were generated from analyzing blanks and samples in full scan mode. Data was acquired using a data-dependent acquisition mode (DDA) and using a top-10 acquisition scheme. Obtritrap resolution was set to 90,000 for the Full Scan and 60,000 for the MS^2 scans and normalized collision energy (%) of 20, 40 and 60 were used. Scan range, RF lens, AGC Target, maximum injection time mode, and microscans remained consistent with those of the full scan method.

NTA Data processing.

Raw data files were analyzed separated using MSDIAL (v5.2)[4]. Raw data files were imported into MSDIAL as profile spectra and data extraction, peak picking, compound identification and alignment was performed. EICs were extracted using mass tolerances of 0.005 Da for MS1 data and 0.01 Da for MS2 data and Cl and Br were considered for data extraction and deisotoping. During peak picking, the minimum peak height was set to 25000 counts and mass slice width was set as 0.1 Da. Data was smoothed with a box width of three scans and at least five datapoints were required for each chromatographic peak. Compound annotation was performed using the following settings: the accurate mass tolerance for MS1 and MS2 were set as 0.0025 Da and 0.01 Da using reference spectra from MassBank of North America in .msp format (MoNA, https://mona.fiehnlab.ucdavis.edu/). The accurate mass tolerance (MS1) and retention time tolerance were set as 0.0025 Da and 0.5 min for comparison to a LC-MS library containing retention times of the authentic standards (.txt library) for confirmation. Data was aligned using 0.0025 Da as MS1 tolerance and 0.5 min as RT tolerance and blank filtering was applied (maximum sample > 5x average blank). Confidence levels of compound annotations were classified using confidence levels 1 (confirmed by authentic standard), level 2 (confirmed by spectral library match), and level 3 (confirmed by spectral library match, but with isomeric structures possible[5]).

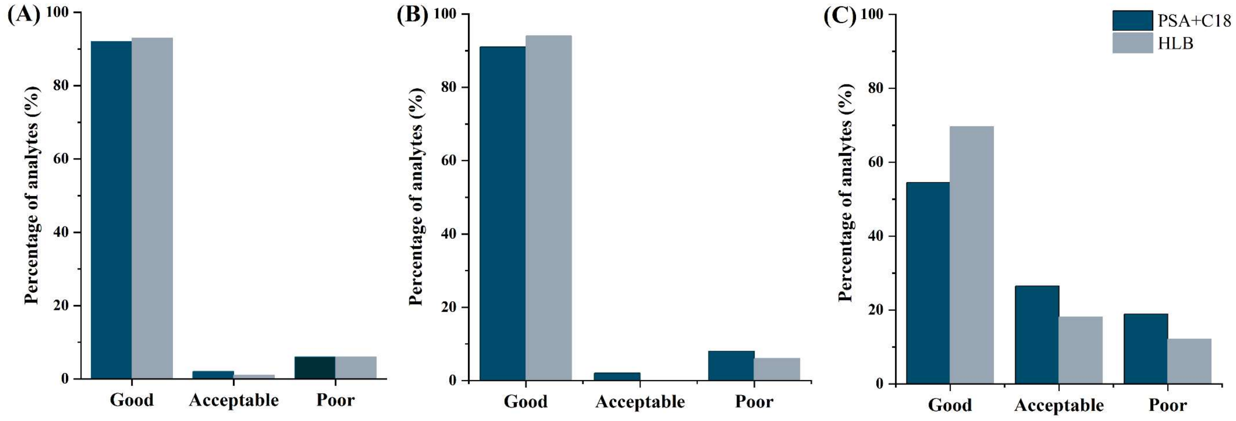

Figure S1. Comparison of two SPE sorbents used for cartridge-based SPE: in-house produced mixed-mode sorbent, PSA (Primary Secondary Amine) + C18 (PSA+C18), and Oasis HLB (Waters). Results show the evaluation of the analyte loss for 66 analytes during sample loading diluted with water (A), SPE washing step using water (B), and extraction recovery (RE, %) for elution with 3% ammonia in MeOH (NH_3/MeOH, 3/97, v/v) as elution solvent (C). "Good" performance was defined as: either analyte loss during loading and washing steps < 10%, or as RE 60% - 140%. "Acceptable" was defined as analyte loss of 10% - 20%, or the RE either 20%-60% or 140%-180%. Any results outside these ranges were classified as the level of "poor".

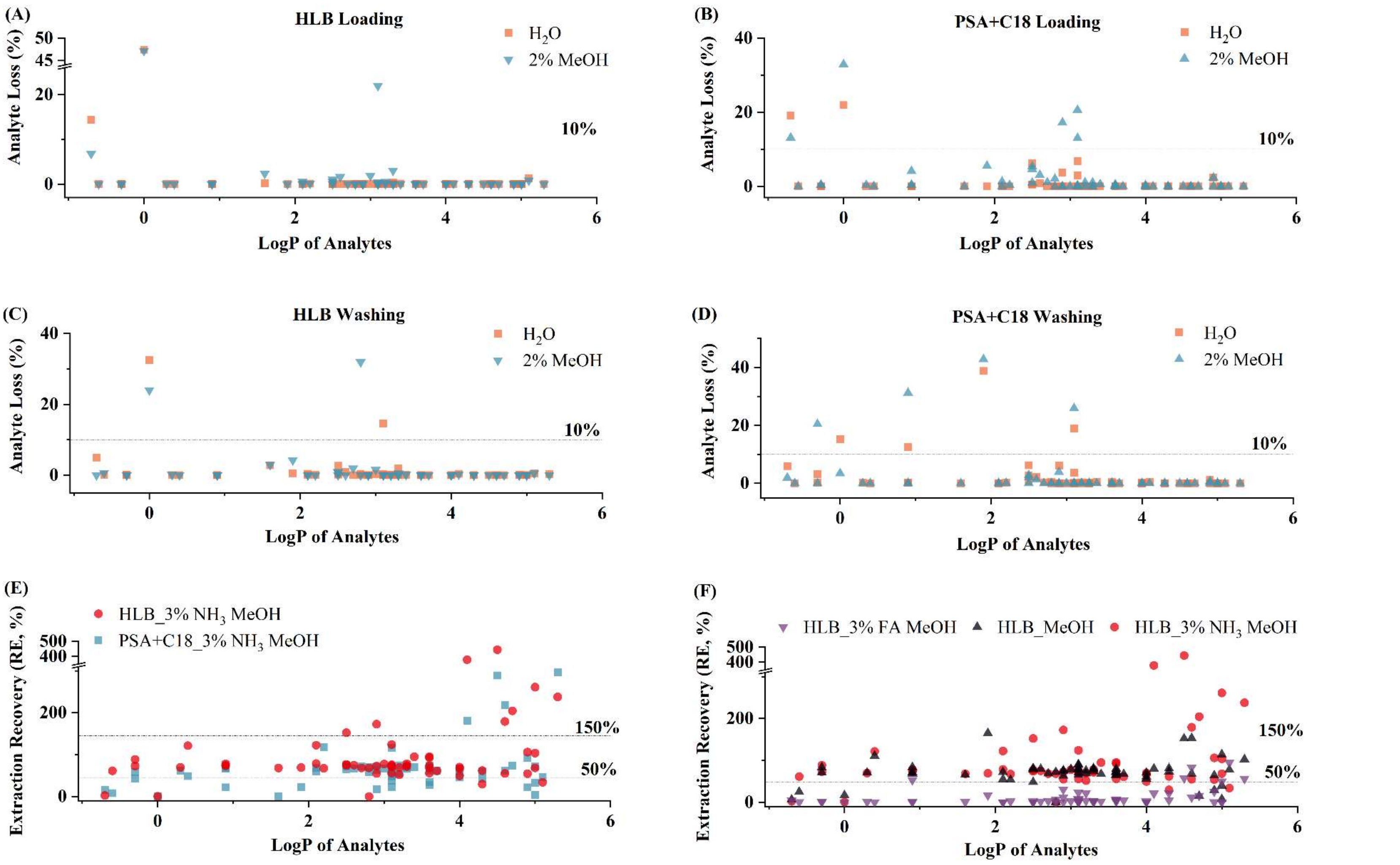

Figure S2 Performance of Oasis HLB (HLB) and in-house produced mixed-mode sorbent PSA (Primary Secondary Amine) and C18 (PSA+C18) used in SPE cartridges. The analyte loss for 66 representative analytes during loading process is shown for HLB (A) and PSA+C18 (B), and losses during washing steps are shown for HLB (C) and the mixed sorbent (D). Extraction Recoveries (RE, %) are presented in panel (E) for 3% of ammonia in methanol (3% NH_3 MeOH, NH_3/MeOH, 3/97, v/v) as the elution solvents, while (F) presents RE using 3% formic acid in MeOH (3% FA_ MeOH, FA/MeOH, 3/97, v/v), pure MeOH and 3% NH_3 MeOH for HLB cartridges.

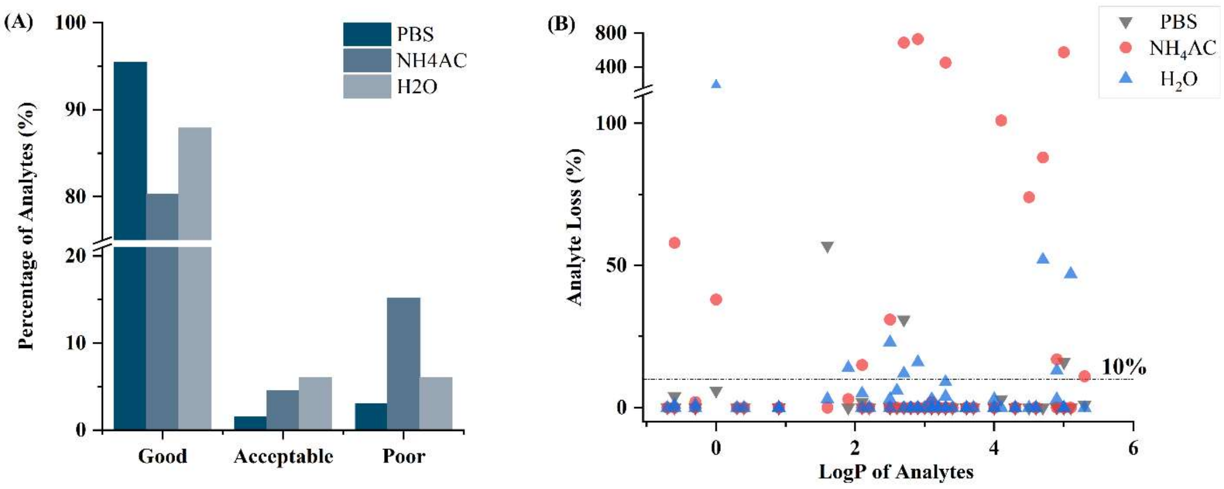

Figure S3. Assessment of three buffers for sample dilution prior to loading onto the HLB cartridge: phosphate-buffered saline (PBS, 200 mM, pH 7.4), ammonium acetate (NH_4AC, 2.5 M, pH 6.0) and pure water. (A) Percentages of analytes out of 66 compounds in "good", "acceptable" and "poor" performance. "Good" performance was defined as analyte loss < 10%, "acceptable" performance was defined as loss 10% to 20%, and larger losses were classified as "poor". The X-axis represented performance levels, and the Y-axis depicted the percentage of compound amounts falling into each level. (B) Analyte losses as a function of octanol-water partition coefficient (LogP) for 66 analytes.

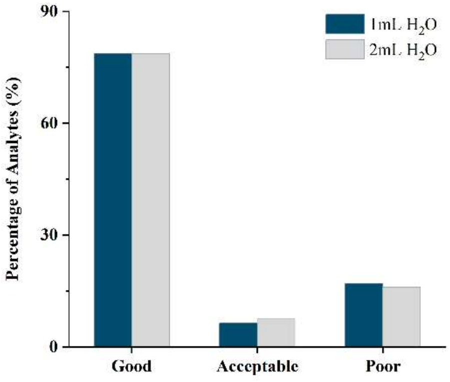

Figure S4 Evaluation of two washing protocols: comparison of 1×1mL and 2×1mL H_2O for washing SPE cartridges and effects, on signal suppression and enhancement (SSE) for 94 compounds in pooled urine using HLB cartridges. "Good" performance was defined as an SSE value between 60% and 140%, "acceptable" performance describes SSE between 20% - 60% or 140% - 180%, and SSE < 20% or SSE > 180% was classified as "poor".

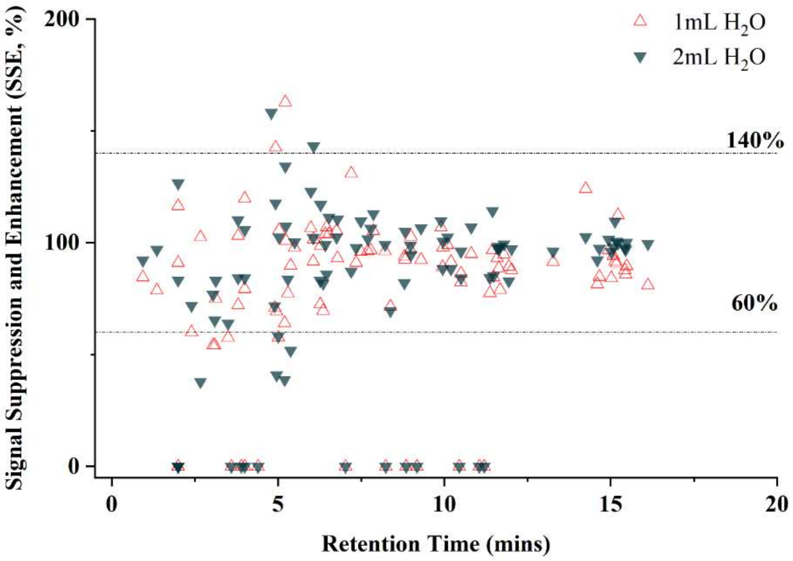

Figure S5. Evaluation of two washing protocols: signal suppression and enhancement (SSE, %) as a function of retention time for 94 compounds in pooled urine using two washing protocols (1×1mL and 2×1mL H_2O) for HLB cartridges. SSE was calculated as the ratio of peak area for postspiked urine to standards in 5% MeOH ($MeOH/H_2O$, 1/1, v/v), with SSE close to 100% indicating negligible matrix effects on analyte intesities.

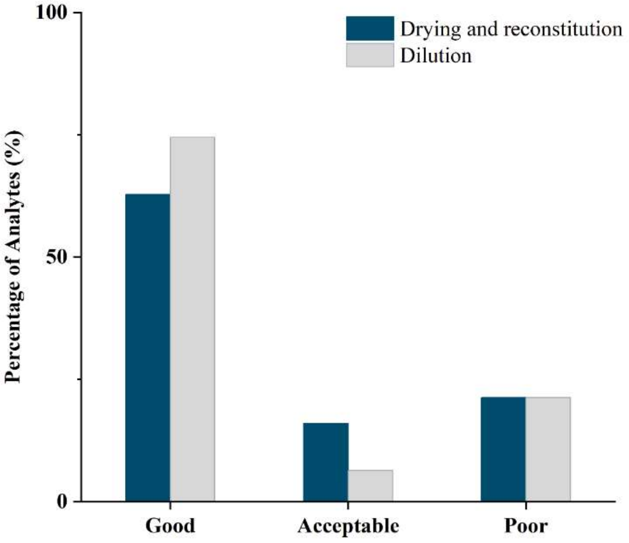

Figure S6 Evaluation of the effect of drying using a vacuum concentrator and reconstitution after SPE process for 94 compounds in pooled urine and comparison to direct dilution of SPE extracts. "Good" performance was defined as RE between 60% - 140%. "Acceptable" performance is defined as RE 20% - 60% or RE 140% - 180%. Any results outside these ranges were classified as "poor".

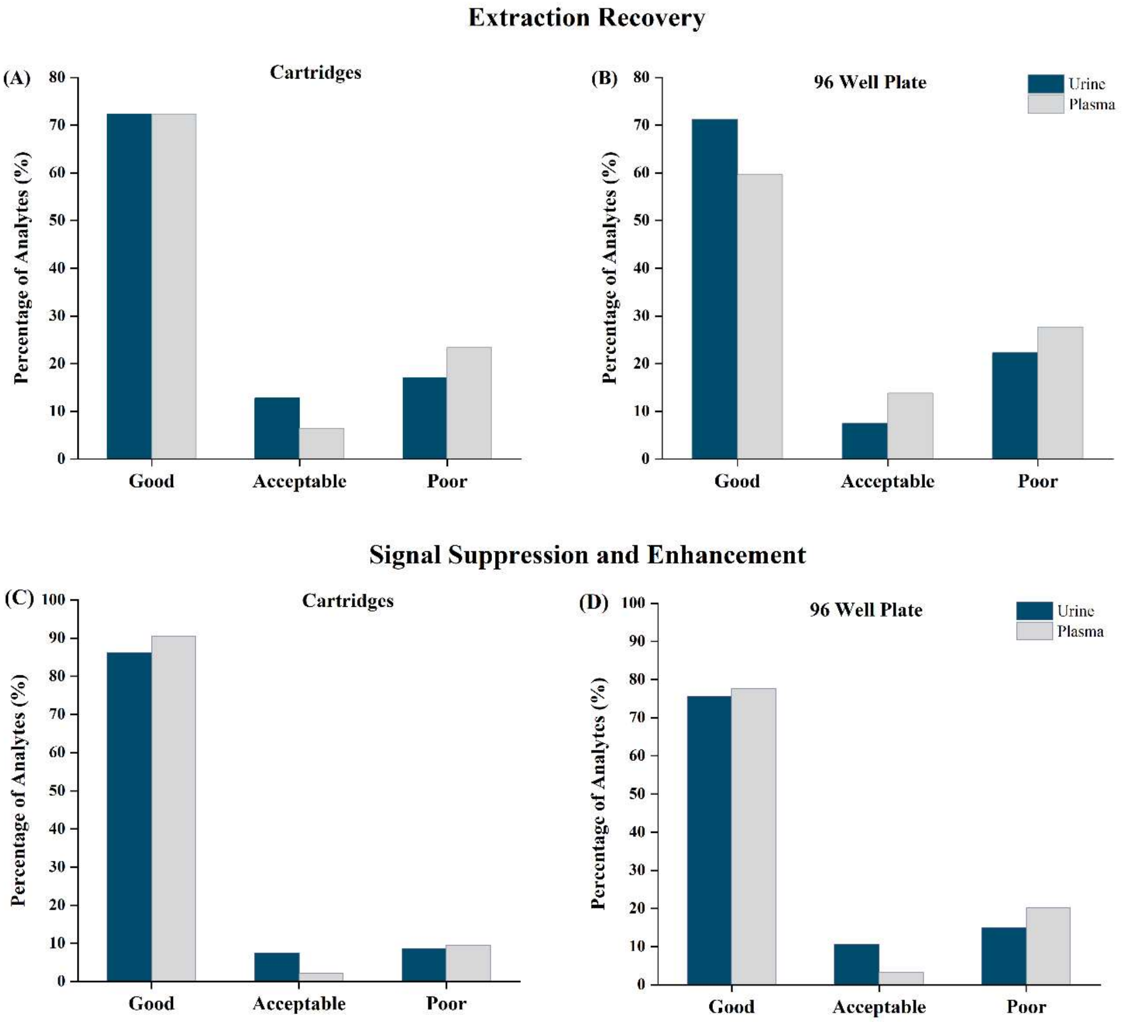

Figure S7. Evaluation of extraction recovery (RE, %) and signal suppression and enhancement (SSE, %) for 94 analytes spiked in pooled urine and plasma samples using SPE cartridges and comparison to results of 96-well plate-based SPE: RE using SPE cartridges (A), RE using 96-well plate-based SPE (B), SSE estimation using SPE cartridges (C) and SSE estimation using SPE 96-well plate-based SPE (D). "Good" performance was defined as RE or SSE between 60% - 140%. "Acceptable" performance is defined as RE or SSE between 20% - 60% or 140% - 180%, whereas all other results were classified "poor".

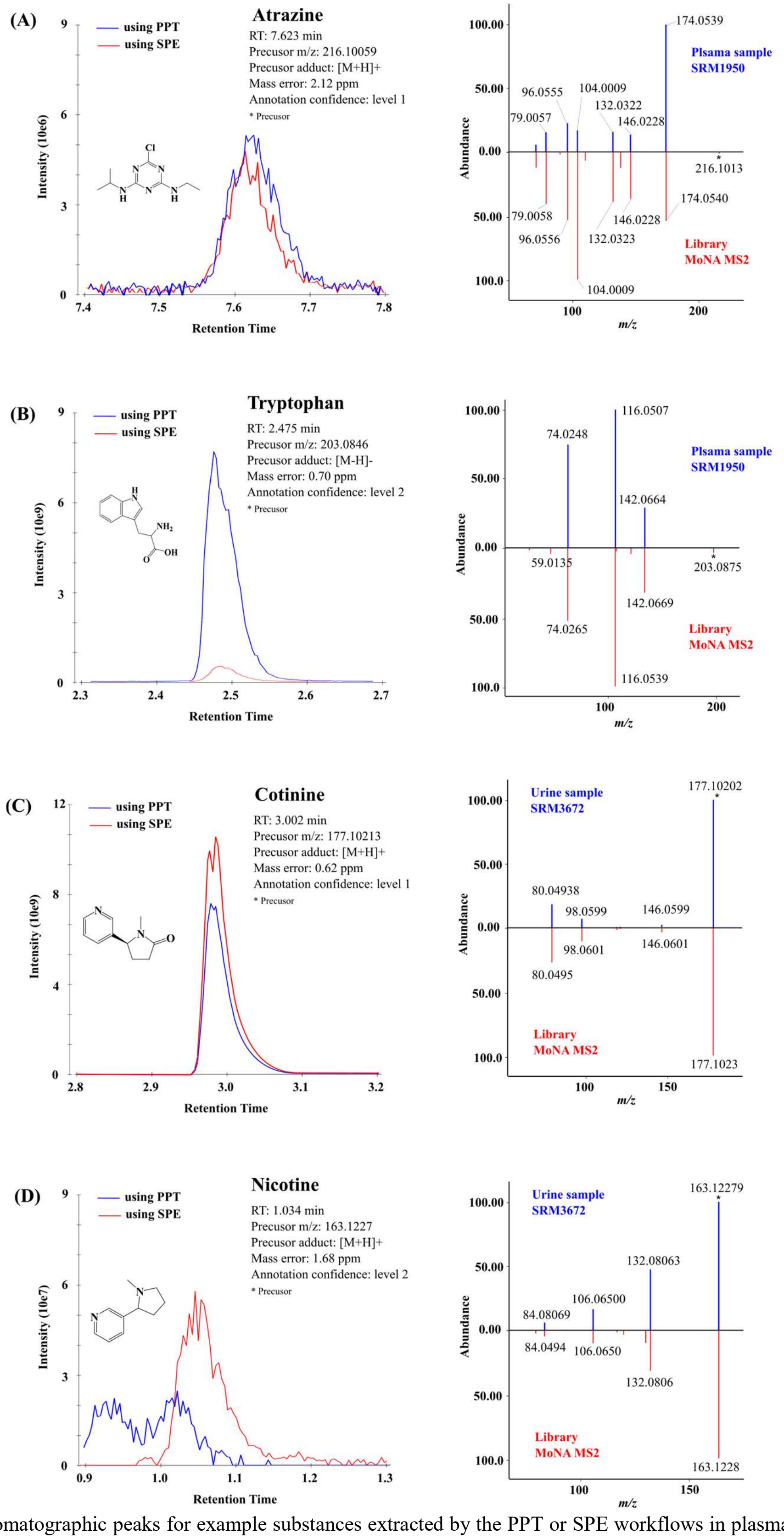

Figure S8. Chromatographic peaks for example substances extracted by the PPT or SPE workflows in plasma samples (SRM1950) in A and B and urine samples (SRM3672) in C and D, annotated at confidence level 1 or level 2 by deconvoluted DDA MS2 spectra matching to the MoNA database.

References

(1) Jamnik, T.; Flasch, M.; Braun, D.; Fareed, Y.; Wasinger, D.; Seki, D.; Berry, D.; Berger, A.; Wisgrill, L.; Warth, B. Next-generation biomonitoring of the early-life chemical exposome in neonatal and infant development. *Nat Commun* **2022**, *13* (1), 2653. DOI: 10.1038/s41467-022-30204-y From NLM Medline.

(2) Matuszewski, B. K.; Constanzer, M.; Chavez-Eng, C. Strategies for the assessment of matrix effect in quantitative bioanalytical methods based on HPLC− MS/MS. *Analytical chemistry* **2003**, *75* (13), 3019-3030.

(3) Preindl, K.; Braun, D.; Aichinger, G.; Sieri, S.; Fang, M.; Marko, D.; Warth, B. A Generic Liquid Chromatography-Tandem Mass Spectrometry Exposome Method for the Determination of Xenoestrogens in Biological Matrices. *Anal Chem* **2019**, *91* (17), 11334-11342. DOI: 10.1021/acs.analchem.9b02446.

(4) Tsugawa, H.; Cajka, T.; Kind, T.; Ma, Y.; Higgins, B.; Ikeda, K.; Kanazawa, M.; VanderGheynst, J.; Fiehn, O.; Arita, M. MS-DIAL: data-independent MS/MS deconvolution for comprehensive metabolome analysis. *Nature methods* **2015**, *12* (6), 523-526.

(5) Schymanski, E. L.; Jeon, J.; Gulde, R.; Fenner, K.; Ruff, M.; Singer, H. P.; Hollender, J. Identifying small molecules via high resolution mass spectrometry: communicating confidence. ACS Publications: 2014.
